# Fostering childhood prosociality and relatedness: the perceived impact of an arts-based philosophical intervention on collaboration, empathy and respect

**DOI:** 10.3389/frhs.2025.1726122

**Published:** 2026-03-04

**Authors:** Maxime Gilbert, Joel Montanez, Zachary D. Fry, Adrianna Mendrek, Catherine Malboeuf-Hurtubise

**Affiliations:** 1 Department of Psychology, Bishop’s University, Sherbrooke, QC, Canada; 2 SMRC et CIUSSS du Centre Sud de L'île de Montréal, Montréal, QC, Canada; 3 School of Psychology, University of Wollongong, Wollongong, NSW, Australia

**Keywords:** art-based interventions, philosophical inquiry, prosociality, relatedness, self-determination theory, socio-emotional development

## Abstract

**Background:**

Fostering prosociality in children, defined as *acting for the benefit of others*, is essential for both individual and collective well-being. According to self-determination theory, satisfying the fundamental need for relatedness plays a crucial role in motivating prosociality. Integrating interventions that combine arts and philosophical approaches reinforces socio-emotional skills fundamental to prosociality by enhancing social awareness and empathy, as well as offering an adaptable and practical approach in academic settings.

**Aim:**

This study examined how children engage with social contexts that call upon socio-emotional competencies, and how these experiences relate to the development of prosociality in art-based philosophical interventions. The study was guided by the following research question: *What are the perceived benefits of an art-based philosophical intervention on children's prosociality and relatedness?*

**Methods:**

This study used a descriptive qualitative design with children in a school setting. Over 10 consecutive weeks, 60-minute workshops combining arts and philosophical inquiry were conducted. Data analysis included group discussions, observations, and 21 semi-structured interviews, with results interpreted through inductive thematic analysis.

**Results:**

The findings indicated that the intervention fostered both connection and engagement among the students: Connection (perceived as emotional resonance) encouraged mutual respect, empathy, and admiration, whereas engagement (which offered comfort, trust, and fostered a sense of belonging) was observed through camaraderie, collaboration, and instances of disengagement.

**Discussion:**

These findings highlight that the development of introspection, self-awareness, and autonomy through artistic and philosophical activities provided a foundation for students to act prosocially, thereby emphasizing empathy, care, and respect toward others.

**Implications:**

The study demonstrates that integrating arts and philosophical inquiry in elementary education fosters prosociality, empathy, and self-awareness, supporting both academic and socio-emotional growth. It offers adaptable strategies for inclusive, cooperative classrooms and highlights implications for curriculum design and policy promoting student well-being and community.

## Background

Prosociality, characterized by *voluntary actions intended to benefit others*, is a fundamental aspect of human behaviour—shaped by evolutionary, psychological, and developmental mechanisms (1, 2). Depending on the theoretical framework, prosociality encompasses cognitive, emotional, and behavioural dimensions, which is demonstrated through altruistic actions, such as helping, sharing and comforting (1, 2). From an evolutionary perspective, prosocial acts ensure group survival through enhanced cooperation and emotional bonds. Despite evolving into individualistic tendencies, these mechanisms still uphold many current social behaviours essential for community living, such as gratitude and trust (3).

Psychological theories provide complementary perspectives on why prosociality persist as a behavioural trait, despite no longer being considered vital for survival. Self-Determination Theory (SDT), developed by Ryan & Deci (23), identifies three basic psychological needs for optimal functioning and well-being: *autonomy*, *competence*, and *relatedness*. Of these needs, relatedness–defined as *the need to feel emotionally connected to others*, acts as a key motivator of prosociality (23). Despite research noting lower levels of relatedness in modern individualistic societies, other literature suggests that they nonetheless remain motivated for seeking, developing, and maintaining strong social bonds (4). This, at minimum, indicates that prosociality continues to remain a fundamental quality of human behaviour and societal group dynamics.

Additionally, research identifies three key traits within prosociality: *empathy*, *collaboration*, and *respect*. Empathy involves recognizing, interpreting, and emotionally responding to another person's feelings and emotions (2). Collaboration is the prioritization of shared goals over personal interests, and includes behaviours such as teamwork, sharing, turn-taking, and valuing others' ideas (5). Finally, respect encompasses recognizing others' opinions, abilities, and contributions, ensuring that everyone is heard fairly and treated equitably (6). Understanding prosociality through these three traits provides a foundation for examining how they can be nurtured in childhood.

### Fostering Prosociality in Childhood

Children have the cognitive and social capacity to act prosocially, as evidenced by their unique motivation to share attention, perceptions, emotions, and information with others (7). Research suggests that children become autonomously prosocial as early as age two, and neither adult directives–nor praise and reinforcement–influence their inclination towards helping others (7). However, although they may innately hold these qualities, young children are not attuned to the complexity of social cues or fully aware of others' emotional states. Within the neuroscientific framework of prosociality, the *experience-expectant process*— that is, the process by which the brain relies on specific inputs to develop efficiently—is continuously refined through social, emotional, and communicative experiences throughout childhood (7). Thus, the full expression of prosocial traits depends on the children's experiences as they grow, such as through interactions with caregivers and peers, which provide the context for these tendencies to be practiced and expanded (7). While prosociality is grounded in innate capacities, its manifestation in children relies significantly on socialization; it is driven by both intrinsic motivators and external influences.

Research on children's social-emotional development indicates that empathy, emotional regulation, and understanding are critical for forming healthy relationships and navigating social situations (2). These findings have led to developments in both school- and home-based programs that are designed to improve these skills. A meta-analysis of nineteen school-based programs focused on social and emotional learning found that interventions aimed at developing these qualities in childhood—along with skills related to empathy—have lasting positive effects on prosocial behaviour (2). Notably, while these programs do not directly target behaviours like sharing or helping, these improvements often emerge intrinsically. Essentially, developing children's social and emotional skills enhances their capacity for prosocial behaviour in social settings. As they grow more aware of their feelings and of those of others, their motivation to act prosocially becomes closely tied to these skills. One practical approach to fostering these capacities in elementary schools is through structured school-based interventions—including creative arts and philosophical inquiry—which offer effective avenues for nurturing prosocial development.

### The role of relatedness in prosociality

Among the three psychological needs, relatedness is the key driver of prosocial behaviour. SDT underscores how intrinsic motivation is optimally developed within interpersonal contexts, particularly in those characterized by secure attachment relationships. For example, a study by Ryan and Grolnick (8) reported lower intrinsic motivation in students when the teacher-student relationship was cold and distant. Moreover, while intrinsic motivation frequently develops within interpersonal interactions, it can also emerge in solitary contexts. This indicates that the foundation of intrinsic motivation lies not solely in the presence of a social environment, but rather in deeper psychological factors such as secure attachment and a sense of belonging (23). This sense of connection and belonging is central to explaining internalized motivations for actions performed for the benefit of others.

Empirical studies also highlight the relationship between relatedness and prosociality. Pavey et al. (27) found that relatedness significantly increased prosocial tendencies in volunteering and donating activities, compared to autonomy and competence, which did not elicit higher prosocial tendencies. Similarly, Shiraki and Igarashi (9) demonstrated that feeling gratitude increased individuals' need for relatedness, which in turn promoted prosocial spending (donating). These findings underscore that having an increased sense of connectedness and community fosters motivation to act prosocially. Within educational contexts, fulfilling the relatedness needs of students is also found to support the further development of social skills, community engagement, and intrinsic motivation to act prosocially (10, 11). Students who engage in prosocial acts also often form stronger relationships with peers and teachers, deepening their sense of belonging. Through diverse experiences, students learn respect, teamwork, open-mindedness, and concern for others (9). Supporting children's sense of relatedness is thus critical for facilitating their prosociality in school settings.

### School-based interventions

One practical approach to fostering prosociality in educational settings is through structured school-based interventions, which provide children with opportunities to practice empathy, collaboration, and respect within supportive social contexts. They have been shown to enhance collaboration, empathy, and relatedness among children (5, 11, 12), while philosophical inquiry encourages critical thinking, personal philosophy development, and socio-emotional growth (6). By combining these approaches, an intervention bridging arts and philosophical inquiry would strengthen socio-emotional competencies and promote prosociality in elementary school students.

#### Artistic interventions

Artistic interventions promote socio-emotional skills essential for prosociality and relatedness, specifically by fostering social bonds, encouraging cooperation, and enhancing community involvement (11, 13). For example, participating in group art activities helps children develop skills like sharing, taking turns, and prioritizing group goals over individual desires (5). By providing opportunities for connection and shared expression, artistic interventions build feelings of belonging and interconnectedness among participants (14, 15). Additionally, they promote empathy, self-regulation, and self-awareness (12), encouraging prosocial responses such as care and compassion for others (15, 16).

#### Philosophical inquiry

Philosophical inquiry, developed by Matthew Lipman and Ann Margaret Sharp in the 1970s, introduces philosophical thinking to pre-college students, enhancing critical thinking and reasoning (17). It fosters children's agency and socio-emotional growth by encouraging awareness of their impact on others within a safe learning environment, allowing reflection on philosophical issues and promoting autonomy (18). Sharp's approach emphasizes the critical role that community plays in meaningful dialogues, teaching children to care for one another by creating spaces for honest discussions free from fear of judgment (19). Evidence shows that P4C can reduce bullying, which can theoretically in turn promote mutual respect, empathy, and fairness (6). Ultimately, philosophical inquiry contributes to children's self-awareness, agency, and overall prosocial development.

## Aim of study

Prosociality fosters cooperation and inclusivity, both of which are central to the well-being of communities and their residents (28–31). Early development of socio-emotional skills presents a critical window in which children's prosocial tendencies and sense of relatedness are more susceptible to the influence of others (2). Building on this, the current study sought to explore how an art- and philosophy-based intervention could contribute to elementary school students' experiences of empathy, collaboration, respect, and relatedness. The intervention combined arts–aimed at supporting collaboration and empathy—with philosophical inquiry–designed to encourage critical thinking and personal philosophy development (5, 6, 11, 12). By integrating the arts and philosophical inquiry, this study examined how children engage with social contexts that call upon socio-emotional competencies, and how these experiences relate to the development of prosociality. Specifically, the study was guided by the following research question: *What are the perceived benefits of an art-based philosophical intervention on children's prosociality and relatedness?*

## Methodology

### Sample

The sample consisted of two groups of sixth-grade elementary school students in the province of Quebec, Canada (*n* = 21, *M*_age_ 11.5). All students in the sample participated in the intervention, during which we conducted observations and facilitated group discussions. Following the intervention, students were invited to share their experiences through semi-structured interviews. Participants were recruited through convenience sampling at a local elementary school in Sherbrooke. Specifically, students from two classrooms of the same grade at this school were invited to participate. Parental consent was obtained, and verbal assent was given by all students. The study received approval from the Bishop's University Ethics Board on December 30th 2023 (File #102681), in compliance with the 2018 Tri-Council Policy Statement: Ethical Conduct for Research Involving Humans (TCPS 2) and the Bishop's University Research Ethics Policy.

### Themes and activities

The intervention was collaboratively developed by a child psychologist (CMH), two master's students (MG and ZF), a clinical psychologist (JM), and a researcher (AM) with dance and movement therapy training. Led by the master's students (MG and ZF) with an undergraduate research assistant, the workshops took place for 10 consecutive weeks, each lasting 60 min, during regular class time. Activities followed a consistent structure: students first engaged in an art workshop and then convened for a philosophical inquiry based on the theme of their creation. As outlined in Table 1, different themes addressed prosociality and relatedness. Artistic workshops utilized various media to maintain engagement, and the topics were designed to progress gradually, fostering deeper discussions.

**Table 1:** Intervention plan outline.

Week	Theme	Activity	Topic Discussion
1	Beauty	Ugly Drawings	Beauty Standards
2	Identity	Lego Self-Portrait	Values
3	Self-Care	Safe Space	Love and Respect
4	Care of Others	Mirror Dancing	Empathy
5	Care of Nature	Photovoice on Nature	Collective Responsibility
6	Emotions (1)	Acting Out Emotions (Dance)	What are emotions?
7	Emotions (2)	Watercolour painting	Why do we experience emotions?
8	Death (1)	Scrapbooking	Personal mortality
9	Death (2)	Sculpture of a Beautiful Death	Bereavement
10	Community	Butterfly Mural	Belonging & Hope

Although the intervention followed a structured sequence of themes and activities, each workshop was intentionally designed to prompt students' reflection. For example, in the *Ugly Drawings* activity during Week 1, children drew intentionally “ugly” images to discuss what can be considered beautiful and ugly, aiming to encourage reflection on individual differences and encourage openness toward peers. In Week 3, the *Safe Space* drawing exercise asked participants to visualize a place where they feel secure, which encouraged discussions on self-care and mutual respect. *Mirror Dancing* invited children to work with peers and mirror one another's movements, giving them an embodied experience of empathy, attunement, and cooperation. Similarly, activities exploring emotions (ex., watercolour paintings) were used to help children reflect on the use and impact of emotions. Across all workshops, philosophical inquiry encouraged children to articulate their ideas, listen to their peers, and collaboratively build meaning on essential topics. Together, these approaches were structured to cultivate prosociality through interactive engagement.

### Data collection

Qualitative data was collected through multiple methods, including audio recordings of group discussions, observation grids and semi-structured interviews with twenty-one students The interviews, guided by open-ended questions (see Table 2), focused on eliciting students' reflections on the intervention and its perceived influence on collaboration, empathy, and respect for others. During the workshops, the research assistant systematically recorded observational notes, which were subsequently reviewed and elaborated on by the master's students (MG and ZF) after each session.

**Table 2:** Examples of questions included in the semi-structured interview.

Section 3: Perceived Prosociality	
Collaboration	What does collaboration mean to you? Can you think about a time during the activities when you worked together with other students? How did that experience make you feel? Were there any specific activities or discussions that made you more collaborative? How have art activities encouraged collaboration in the classroom?
Empathy	What does empathy mean to you? Can you think about a time during the activities when you felt empathy? How did that experience make you feel? Were there any specific activities or discussions that made you understand others’ feelings? How have the activities we did together helped you understand others’ feelings?
Relatedness	Can you tell me what the word community means to you? Was there a time during the activities when you felt a sense of community in the classroom? How did that experience make you feel? Were there any specific activities or discussions that made you feel more connected to your classmates? How did those activities promote a sense of community in the classroom?
Respect	Can you tell me what respect means to you? Was there a time during the activities when you appreciated someone else's art or ideas? How did that experience make you feel? Were there any specific activities or discussions that made you more respectful towards your classmates? Why?

### Data analysis

Data was analyzed using a thematic analysis (inductive and deductive) using the MAXQDA24 software to identify and categorize patterns in participants' interview responses. The coding process began with open, line-by-line inductive coding of the transcripts by the first author to generate preliminary codes. These initial codes were used to develop a draft codebook that included code definitions (see Table 3). Initial coding was then cross-validated with two research assistants (undergraduate psychology students). After this process, the team met to compare coding decisions, discuss discrepancies, and refine code definitions through consensus. The identified themes were then compared with data from observation grids, artwork, and group discussions to support patterns. The final themes were reviewed by two clinicians (CMH & JM), and relevant examples were extracted to support the codes and generate study results.

**Table 3:** Summary of codes with definition.

Theme	Sub-Theme	Definition
Connection	Respect	*Being mindful and honouring others’ inner experiences with due regard.*
	Empathy	*Sharing and understanding others’ feelings and emotions towards a sensitive situation.*
	Admiration	*The recognition of other's talents and ideas as impressive and original.*
Engagement	Camaraderie	*Preference for intimate social groups where trust and comfort are already established.*
	Collaboration	*Collective participation in sharing ideas and opinions for the greater sense of the conversation.*
	Disengagement	*Holding low regard and undermining other's emotions and experiences through negative behaviours.*

### Positionality statement

The lead researcher of this project approached this study with an interest in arts-based learning and prosocial development. My training in qualitative research and my commitment to fostering supportive, engaging classroom environments informed both the design of the intervention and my interpretations of student participation. Although I did not have a prior relationship with the participating students, my role as an on-site adult researcher may have influenced how students engaged during sessions, particularly in moments of vulnerability or self-expression. To mitigate these influences, I worked collaboratively with the classroom teachers and the other researchers who were also present during data collection (see Methods). I also engaged in ongoing reflexive note-taking with my team to examine how my assumptions and interpersonal positioning shaped the analytic process. These practices supported a transparent, critically reflexive approach to understanding the diverse experiences that emerged during the intervention.

## Results

The analysis revealed two overarching themes, connection and engagement, within the arts-based philosophical intervention that emerged within the group. Connection–marked by emotional resonance, noted by the closeness and mutual understanding, is associated with respect, empathy, and admiration among peers. Engagement–as observed in the camaraderie and collaboration subsection)—were shaped by group dynamics. Although students generally described experiences of strong connection and engagement within the intervention, the data also revealed occasional instances of disengagement that emerged in response to group dynamics and emotionally sensitive content. These instances, while less frequent, provided important contrast and nuance to the overall patterns of participation.

### Connection

Throughout the intervention, students mentioned having sensitivity to the emotions of others, especially when discussing personal and sensitive topics like safe spaces, death, and vulnerability (see Table 4). Hearing peers share personal stories helped students recognize their own and others' emotional experiences, fostering a classroom culture rooted in respect, empathy, and admiration. Many became more sensitive, practicing active listening and openness while withholding judgment. These conversations encouraged students to see their peers in a more genuine light, aligning with deeper emotional connections and acceptance. Observations similarly noted that verbal sharing and artistic expression revealed students' vulnerability, suggesting stronger bonds and creating a supportive environment. In addition, creative activities provided students with the opportunity to admire one another's talents, further indicating mutual appreciation and connection. This combination of emotional openness and shared admiration suggests a more positive and respectful classroom dynamic where students feel seen, valued, and connected.

**Table 4:** Illustrative quotes of connection from the intervention.

Connection	Illustrative Quotes
Respect	“When we were gonna talk about death…it's a topic you have to be respectful of because you don't wanna hurt anyone's feelings” (student) "When we talked about death… I just wanted to be more respectful so that people didn't feel worse.” (student) “Well, when we were talking about death, I felt like they respected me a bit more because I had something to say and I had an experience, so they listened to what I had to say.” (student) “I’d say, for me personally, I think it was like, the talking emphasized more respect because it was like respecting other people when they're talking. Not like, saying that, your idea is better than theirs and stuff. And listening to everybody” (student) “Well, like some ways it's kind of like, I think when other people like open up and other people can like also respect like their saying or ideas.” (student)
Empathy	“I think the death one because it, like, helps me understand more for like my friends and stuff to see what they are going through. And like, I can help them if they need help and stuff.” (student) “Like for the death one like somebody's dog has passed away not that long ago so- I could put myself in his shoe because like, I lost a dog, and I was sad…” (student) “I started understanding why people feel so sad all the time in class…Because I understood how they felt….” (student) “Like when we were talking about death again. Like, there's people that didn't really like it. Like they went out to class and like I went. If I put myself in their shoes, I maybe understanded them because, like, maybe some close people died not long ago. And they still think about them.” (student) “The discussions helped us because, uh, have more empathy because you're getting more into personal things instead of just like backgrounds. And when it's more personal, you start to get more serious and understand what they're going through.” (student) “I think the discussions help me a lot understand, like what other people might be going through. Like, like feel their opinions and stuff on others things” (student) “Discussion is definitely providing more empathy because you get to listen to people talking about a specific topic. And you get to show empathy towards what they're talking about.” (student)
Admiration	"I liked the photography one because…I got to see…what you see from their eyes…you can create something beautiful out of just…a small playground.” (student) “The discussions when they like express their feelings about things. That they expressed why they were upset about it or like a quote that they had in their little drawing… And we all were like connected at that time because we were like “Ohh, I like yours” “Oh my gosh, it's so funny and cool.” (student) “When we were doing like the activities like the… the crafts and stuff. I think it was like a lot of people around me are really good at art and I respected and like thought there's were really cool.” (student)

### Engagement

Students' engagement was strongly influenced by camaraderie and collaboration (see Table 5). Camaraderie, often found in pre-existing friendships, offered comfort, trust, and a sense of belonging between participants. Many participants felt more at ease when surrounded by friends, often seeking ways to remain in familiar groups, especially during art activities. Smaller group settings–as opposed to larger discussions—provided a safer space for expression and creativity. This familiarity helped mitigate anxiety around sensitive topics and encouraged greater involvement and sharing during the intervention. This comfort extended to group discussions and creative endeavours, where smaller group settings allowed for collaboration. Students were observed enthusiastically brainstorming responses to philosophical questions and initiating thoughtful discussions on topics like identity and the future. The blend of trust and teamwork fostered a sense of belonging and mutual respect, creating a classroom culture where students felt safe to engage, contribute, and collaborate openly.

**Table 5:** Illustrative quotes of engagement from the intervention.

Engagement	Illustrative Quotes
Camaraderie	Yeah. The last activity we did when the butterfly was with all my friends, I felt like I found my people.” (student) “Because we hang out with each other every single day. So then, like, we just all belong together since kindergarten…” (student) “If I wasn't sure what to do, I'd ask my friend and then we’d help each other and stuff.” (student) “Smaller groups are fun, especially when you’re put with the people you are good friends with.” (student) “When I’m in the smaller groups I feel more like… not energetic but like more… comfortable. Like I feel more comfortable just in the smaller groups.” (student)
Collaboration	“The more the people, the better the discussion and I think most of us shared a good part of stuff so I think we all- I think we all kind of worked with each and every one of us.” (student) “Yeah, when we would sit with some people, I feel like they gave us some ideas so it would really help us build our stuff.” (student) “Yeah, like when we're doing our art, like at the Table 1 was at, we were talking, and we helped each other….A lot of people were sharing their ideas… it did feel like it was collaboration.” (student) “I tried to give ideas and then I saw that throughout the weeks people started adding more and more because they knew that, you know, it was a safe place…where they could talk.” (student) “When we were talking about like, let's say like, death or something, we had to like, collaborate and like communicate to like be on the same page and stuff…” (student)
Disengagement	“When I'm talking like I'm trying to say something and someone cuts me off. It's like disrespectful, so like wait for your turn when something like- let's say I'm doing something and somebody just comes and likes, just move out the way then that's disrespectful. So, you’re like you have to be kind and respectful for us to be respectful back.” (student) “Like sometimes when we're discussing about why we made it, we didn't really feel respected because some people were judging…” (student) “Sometimes when you want to let your emotions out you just can't because some people will make fun of you.”/ “Sometimes, people make fun of you for your emotions,” (Students from Group 1, Week 8) “MG: Do you feel comfortable opening up when you’re in a group? Student: No- Because I don't like a lot of people… Yeah.. I don't trust people in here…” (Student from Group 1, Week 10) “Yes, because a lot of people make fun of each other and then it hurts our feelings… Like when we were talking about death and then I started laughing because of [a student name] said something funny. And then [a student name] started like saying like you can't laugh to something being dead. I kind of understood that. But at the same time I didn't because like people deal with that- well they deal with that by laughing.” (student) “It it made me understand that, you know, I mean I've always been focused on other people's feelings. And I feel like, I mean, when I talk to my friends and I ask them, like, how are you and stuff like that? They say good. And they don't really ask it back to me. I feel like, I mean, I'm a little sad…because, you know, if like if I say no to my friends just gonna be like, ohh. And then, you know, we continue on with the conversations… Like unless like you have a therapist or someone that you know, you feel safe to talk to then you know you could say no and someone would actually like take the time to understand why.” (student)

Despite overall positive engagement, instances of disengagement emerged, often linked to a lack of respect or empathy. Some students expressed discomfort when peers dismissed or undermined their emotional experiences, particularly during sensitive discussions. Disrespectful behaviours such as laughing and teasing were sometimes rationalized as coping mechanisms and made the space feel emotionally unsafe. Observational data revealed issues like talking over others, damaging artwork, gossip, and conflict, all of which disrupted the safe environment and limited meaningful participation. These moments of disrespect compromised trust and discouraged vulnerable engagement for some students. These patterns suggest that engagement and disengagement coexisted within the group, reflecting students' varying levels of comfort and readiness to participate, particularly during activities requiring emotional vulnerability.

### Appreciation of the workshops

Students reflected on and shared an overall positive reaction to the intervention. Workshops addressing sensitive topics—particularly those related to death—emotions, and creating safe spaces- were consistently highlighted as the most memorable to them. While the discussions on death were both meaningful and challenging, they were noted for significantly fostering empathy and a sense of relatedness. The play-dough activity on safe spaces, scrapbooking on death, and watercolour activity on emotions were among the most appreciated for highlighting self-expression and connection. Despite some discomfort, students mentioned that these sessions were seen as critical to developing their sense of prosociality through the socio-emotional skills acquired during the intervention. Students shared the following statement concerning their impression of the intervention on their prosociality:

“Well, the death, yes, wasn't fun like at same time, it didn't make me think about it. And like the rest were also like that… like it could help me with something” (student).

“Some people are like shy to talk about it and don't really like it. So, like it helps people talk more- like express more of their feelings” (student).

Students also valued the intervention for its novelty and emotional depth. Many appreciated the creative freedom, the use of diverse art mediums, and the chance to express themselves in ways not typically available in school. The intervention was widely regarded as fun, expressive, and contributing to their personal growth. Students shared their overall appreciation of the workshops:

“It was fun and most of our classes we don't get to do like the stuff that you guys did.” (student)

“It was quite exciting doing the art activities and it was really fun to express emotions through the art activities” (student).

“Yeah, I found it fun. I found it, I found it different to talk about things that aren't school related. I found it very interesting and overall, not to say that I liked it, but it was relieving I guess you could say” (student).

## Discussion

This study aimed to explore the perceived benefits of an art-based philosophical intervention on children's prosociality and relatedness. Following a 10-week intervention combining arts and philosophical inquiry, 21 students shared their thoughts and perspectives on their perception of the intervention on their prosociality, revealing two main themes: (1) *a sense of connection*—marked by respect, empathy and admiration, and (2) *engagement in the classroom*—marked by collaboration, camaraderie, and instances of disengagement. In this discussion, we analyze these findings by building on the affective dimension of the intervention, specifically corresponding to introspection and self-awareness, the role of autonomy, and the expression of empathy, care, and respect through this process.

### Introspection and self-awareness

The development of socio-emotional competencies—such as empathy, care, and respect—is preceded by the ability to recognize and interpret the meaning of one's own emotions. Levy and Farber (20) defined introspection as the inward focus on one's private emotional life, whereas self-awareness is the ability to evaluate one's behaviour in light of internalized social norms (32). Together, these processes allow individuals to navigate emotions authentically, regulate their behaviour, and understand the impact of their actions on others.

Within this intervention, artistic creation and philosophical inquiry were associated with an opportunity for emotional exploration. Workshops that were more emotionally heavy offered space for reflection, helping students uncover sensitive aspects of themselves. As mentioned in the results, students discussed a particular sensitivity to others' emotions during discussions that encouraged vulnerability. The exposure to such contexts offered an opportunity for self-exploration by offering a safe space to reflect on their emotions. The approaches used in this intervention are well-documented as helpful in fostering introspection and self-awareness. Brazzelli et al. (21) emphasized this same point, highlighting the role of dialogue about internal states in developing emotional understanding. Additionally, Brouillette (7) noted how the arts are found to promote deeper introspection and responsiveness to emotion.

The link between introspection and prosociality is also highlighted by Miller and Verhaeghen (22), who identified the cognitive, emotional, and motivational dimensions of compassion, which is defined as the sensitivity to others' suffering. Their findings emphasized that compassion and the desire to alleviate hurt are strongly linked to mindfulness, which encompasses both self-awareness and introspection. To be genuinely prosocial, individuals must first develop an understanding of care on their own terms. In our study, this phenomenon was observed through the connection fostered among students. Students recognized the importance of empathy during emotionally-charged conversations, practicing active listening and withholding judgment towards others. They recognized that, in their shoes, they would appreciate the same attitude from others.

### Autonomy

During these workshops, the emotional reflection prompted by introspection and self-awareness offered students a sense of autonomy over their behaviour (component of the self-determination theory by Deci and Ryan). Autonomy is the capacity to regulate one's actions and decisions in accordance with one's own personal and social standards (23). When autonomy is prompted, individuals reflect on their moral identity and adjust their actions based on their own social and ethical standards (32). Moreover, according to self-determination theory, the development of autonomy is noted as a key motivator of sustained prosociality; that is, individuals whose actions stem from intrinsic motivation are more likely to act compassionately on a consistent basis over time (24).

In this intervention, philosophical inquiry was recognized to nurture autonomy by encouraging students to form and trust their own judgments. Rather than imposing behavioural norms, the process allowed them to reflect critically on their thoughts and values. Previous literature has explored the use of philosophical inquiry as a tool to nurture autonomy in children. Biggeri and Santi (18) argued that philosophical inquiry builds children's self-confidence and orientation toward self-determination, while our research team (25) previously found it helps students feel ownership over their learning and beliefs. This sense of autonomy motivated students to act in ways that were empathetic and respectful. Guided by their internal standards, they engaged in prosocial behaviour not out of obligation, but because they genuinely valued the well-being of others.

The presence of occasional disengagement also offers insight into the relational conditions required for prosocial development. According to SDT, relatedness and emotional safety are prerequisites for sustained motivation (23). In this study, disengagement often arose when students felt unheard or embarrassed, suggesting temporary compromises to the sense of relatedness necessary for open dialogue. These moments underscore that prosociality is not only cultivated through positive connection, but also shaped by how students navigate ruptures in respect and empathy. Ensuring structures that restore safety may therefore be essential for maximizing the benefits of arts-based philosophical interventions.

### Empathy, care and respect

Students' experiences during the intervention also led to noticeable recognition in empathy, care, and mutual respect. By validating their own and others' emotions, practicing active listening, and withholding judgment, students demonstrated greater emotional attunement to peers. Vulnerable exchanges during workshops helped forge deeper emotional bonds, as represented by more respectful and empathetic behaviour.

Philosophical inquiry played a central role in this process. Sharp emphasized the high potential for philosophical inquiry to tap into emotional life and cultivate care for others, a view echoed by Morehouse (26), who argued that it promotes reflection on others' lived experiences. Other research has also demonstrated its role in cultivating prosocial behaviours such as mutual respect and care (6, 10). Likewise, the artistic workshops fostered empathy and connection among the students. By offering space for creative self-expression, students developed mutual appreciation and admiration for one another's ideas and talents. These shared creative experiences indicated a deepening emotional connection. Similar outcomes have been documented by Kou et al. (12) and Tymoszuk et al. (15), who found that arts engagement fosters lasting empathy through meaningful social interaction.

Together, introspection, self-awareness, and autonomy connected with the development of empathy, care, and respect between participants in the intervention. Through philosophical inquiry and artistic expression, students gained insight into their emotions and moral identity, which in turn empowered them to act prosocially towards others. The intervention created a space where emotional honesty, reflection, and mutual recognition are connected, represented as prosociality rooted in both self-understanding and social connection.

### Ethical considerations

The study raises several important ethical considerations when working with children in emotionally charged contexts. One key issue was that the intervention created opportunities for students to share personal feelings and experiences, whether through artistic expression, small-group conversations, or whole-class discussions. This placed a clear responsibility to maintain a safe and supportive environment where confidentiality, trust, and respect needed to be upheld. Because the activities often involved introspection and self-expression, particular care had to be taken to ensure students' emotional well-being. In this study, the school where the workshops were led offered counselling services for the children during and after class. Students were also encouraged to share their difficulties with people they trusted, such as their teachers, peers, or the Master's students hosting the workshops. We acknowledge that while such activities can be meaningful, they may also bring to the surface difficult emotions to the surface (e.g., when discussing death). It was therefore important to closely monitor students’ reactions and be flexible in redirecting activities if students showed too many signs of discomfort. These could include being reluctant to participate in the discussion (such as distracting the class or disengaging by looking away), having strong emotional reactions to certain topics (like discomfort), and leaving the classroom to avoid the conversation or experience emotions in private. The gradual progression toward more sensitive topics was especially valuable in this project, as it not only helped build depth but also gave students time to develop trust in the process before engaging in conversations calling for more vulnerability.

Despite generally positive experiences, instances of disengagement offered insight into the relational challenges of implementing arts-based, philosophical interventions such as this one. Disengagement most often appeared when respect or empathy within the group was lacking. For instance, behaviours such as laughing, teasing, and talking over others disrupted the atmosphere of trust that the workshops needed. Observational data also highlighted more overt forms of disconnection, including intentionally damaging one's artwork, gossip, and interpersonal conflict. Collectively, these patterns of disengagement highlight the importance of shared respect and structured support for students who may struggle to express emotions in groups. Recognizing these challenges contributes to a more nuanced understanding of how such interventions function in real-world school contexts and can inform strategies to facilitate their future implementation. Overall, while art-based philosophical interventions appear to offer rich opportunities for socio-emotional growth, their implementation requires careful ethical attention.

### Limitations and directions for future studies

This study is subject to limitations that should be taken into account when interpreting the findings. The limited time allocated (60 min in total, including 30 for artistic creation) may have reduced the depth of engagement in the creative process, potentially limiting their perceived effectiveness in reflecting emotionally. Rather than moving quickly from one project to another, students could have multiple sessions to develop and refine the same project. For example, one session could be devoted to creation (30–40 min), followed by a discussion of the work completed, with the next session continuing the reflection. This approach would provide more space for self-expression and thoughtful engagement while avoiding sessions that are entirely focused on art-making. Of course, the duration and structure of activities should be tailored to the age and developmental level of the students, with shorter periods for younger children and longer ones for older students.

In addition, the homogeneity of the sample restricts the capacity of the results to conclusively generalize to a broader population of children. The intervention took place within a single school context and focused on one grade level, which restricts applicability to children of different ages or educational settings. Factors such as age, developmental stage, cultural background, and school environment influence how students respond to such interventions. The relatively short overall duration of the program also prevents conclusions about the sustainability of observed changes in prosociality. Future studies should therefore consider larger and diverse samples, including different elementary programs, and a longitudinal design to examine whether these perceived effects endure over time and across contexts.

Lastly, participants from this study may have been subject to social desirability bias because the master's student who facilitated the intervention also participated in conducting the semi-structured interviews and the data analysis. Although common in participatory research, this dual role may have influenced how children responded during interviews. We attempted to mitigate this through standardized interviews, external code-checking by research assistants not involved in the intervention, and independent review of the thematic analysis by two clinicians.

### Implications

This study contributes to understanding how the arts and philosophical inquiry can foster prosociality and relatedness in elementary education. The findings suggest that creative and reflective activities relate to self-awareness, empathy, and respect, highlighting the importance of addressing both academic and socio-emotional development in schools. For educators, the study provides practical strategies for creating inclusive and cooperative classroom environments. The intervention offered students opportunities to express themselves, engage with their peers, and reflect on their personal values. The adaptability of these approaches allows them to be implemented across different grade levels, learning styles, and educational contexts. At a broader level, the findings have implications for curriculum design and educational policy. Integrating structured opportunities for creative and reflective engagement into school programs may support the development of prosociality, aligning with a stronger student well-being, and a sense of community within classrooms.

## Conclusion

In all, the value of arts-based philosophical interventions revealed to foster socio-emotional competencies relevant to the development of prosociality in children. The intervention highlighted how fostering introspection, self-awareness, and autonomy is an essential tool in shaping empathy, care, and respect. By engaging in philosophical inquiry and arts, students were able to explore their emotions, reflect critically on their values, and develop a stronger sense of agency over their behaviours. These processes fostered prosociality grounded in compassion and mutual respect.

In all, art-based philosophical interventions hold important implications in fostering socio-emotional competencies relevant to the development of prosociality in children. The intervention highlighted how introspection, self-awareness, and autonomy are essential tools in cultivating empathy, care, and respect. By engaging in philosophical inquiry and arts, students were able to explore their emotions, reflect critically on their values, and develop a stronger sense of agency over their behaviours. These processes are connected to prosociality, which is grounded in compassion and mutual respect. Finally, the study lays a foundation for future research on fostering prosociality and relatedness in the classroom. Subsequent research could investigate the long-term effects of these interventions, explore variations across different cultural and developmental contexts, and identify best practices for implementing them across various educational settings.

## Data Availability

The datasets presented in this article are not readily available because confidentiality. Requests to access the datasets should be directed to Maxime Gilbert, mgilb081@uottawa.ca.
